# Clinical characteristics and therapeutic analysis of 51 patients with Marjolin's ulcers

**DOI:** 10.3892/etm.2015.2699

**Published:** 2015-08-24

**Authors:** RUI SHEN, JINMING ZHANG, FENGGANG ZHANG, YONGJUN DU, WEIQIANG LIANG, LUSHENG XU, XUELIANG DU, PING CHEN, XIAODONG CHEN

**Affiliations:** 1 Department of Plastic Surgery, The Second Affiliated Hospital of Sun Yat-sen University, Guangzhou, Guangdong 510120, P.R. China; 2 Department of Plastic Surgery, The Affiliated Foshan Hospital of Sun Yat-sen University, Foshan, Guangdong 528000, P.R. China

**Keywords:** Marjolin's ulcer, squamous cell carcinoma, sentinel lymph node, chronic skin ulcer, metastasis, ^18^F-fluorodeoxyglucose positron emission tomography-computed tomography

## Abstract

Marjolin's ulcers, which are epidermoid carcinomas arising on non-healing scar tissue, may be of various pathological types, including squamous cell carcinoma. The pathogenesis of squamous cell carcinoma arising in an ulcer differs from that of the primary cutaneous squamous cell carcinoma. This squamous cell carcinoma is aggressive in nature, and has a high rate of metastasis. Between January 2001 and September 2013, 51 patients with Marjolin's ulcers were admitted to the Departments of Plastic Surgery of the Affiliated Foshan Hospital and the Second Affiliated Hospital of Sun Yat-sen University. The ulcers included 43 cases of squamous cell carcinoma, six of melanoma, one of basal cell carcinoma and one of epithelioid sarcoma. The clinical data of these patients were retrospectively analyzed. Patients were followed until mortality. Among the patients with squamous cell carcinoma, 30.23% exhibited sentinel lymph node metastasis and 11.63% had distant metastasis. Among the patients with melanoma, 66.67% had sentinel lymph node metastasis and 33.33% had distant metastasis. Sentinel lymph node metastasis was successfully detected in 11 patients with Marjolin's ulcer using ^18^F-fluorodeoxyglucose positron emission tomography-computed tomography and B-mode ultrasound guided biopsy. Squamous cell carcinoma was often treated by extended resection and skin grafting or skin flap repair. Patients with deep, aggressive squamous cell carcinoma of an extremity and sentinel lymph node metastasis underwent amputation and lymph node dissection. This treatment was also used for melanoma type Marjolin's ulcers.

## Introduction

Jean Marjolin first described malignant change arising in a skin ulcer in 1828. This condition was subsequently described by Smith in 1850 and Da Costa in 1903 (1,2). As these ulcers have not been extensively studied, the mechanisms underlying carcinomatous change remain unclear. Marjolin's ulcers are frequently induced by scarring following deep burns caused by hot ceramic, metal or soil (3–5). Marjolin's ulcers are usually considered to be highly aggressive tumors, with a rapid rate of regional metastases. Radical excision is the primary treatment option; however, there is currently no consensus regarding the efficacy of lymph node dissection. Marjolin's ulcers are typically associated with a poor prognosis, and may be life threatening. As living standards improve, the incidence of Marjolin's ulcers should gradually decrease. Although there are few reports of patients with Marjolin's ulcer in China (6), this is not a rare disease, even in the relatively well-developed Pearl River Delta region. The Departments of Plastic Surgery of the Affiliated Foshan Hospital (Foshan, China) and the Second Affiliated Hospital (Guangzhou, China) of Sun Yat-sen University, located in the Pearl River Delta region, treated 51 patients with Marjolin's ulcers between January 2001 and September 2013.

## Materials and methods

### 

#### Patients and data collection

Fifty-one patients who were treated for Marjolin's ulcers between January 2001 and September 2013 were retrospectively reviewed. Follow-up was continued for more than one year. The diagnoses were verified by incisional biopsies in all cases. The specimens received by our laboratory were fixed by formalin and processed using routine hematoxylin and eosin staining. Data collected included age, gender, time from initial ulceration to carcinomatous change, cause of initial ulceration, history of ulcer treatment, surgical treatment and follow-up results. The associations between pathological type and metastasis and between the location of squamous cell carcinoma and metastasis were analyzed. Eleven patients with deep, aggressive squamous cell carcinoma or melanoma and suspected sentinel lymph node metastasis underwent ^18^F-fluorodeoxyglucose positron emission tomography-computed tomography (PET-CT) and B-mode ultrasound-guided biopsy, with a 100% accuracy rate, for the detection of sentinel node metastasis. This retrospective study was approved by the ethical review boards of the participating instututions and written informed consent was obtained from all patients or their next of kin.

## Results

### 

#### Patients

The 51 patients with Marjolin's ulcers included 22 males (43.14%) and 29 females (56.86%) with a mean age of 64.15 years (range, 32–89 years). The mean time from initial ulceration to diagnosis of squamous cell carcinoma was 13.42 years (range, 6 months-54 years) and to diagnosis of melanoma was 2.47 years (range, 3 months-10 years). One patient developed epithelioid sarcoma after two years and one developed basal cell carcinoma after three years. Squamous cell carcinomas were located on the lower limb in 31 cases, the upper limb in seven cases, the head in four cases and the chest in one case. The six cases of melanoma were all located on the foot. One case of basal cell carcinoma was located over the occipital area and one case of epithelioid sarcoma was located on the foot.

The underlying injury causing ulceration was a burn scar in 35 cases and a traumatic wound scar in 16 cases. Ulceration was usually present for a long time prior to carcinomatous change. The non-healing of ulcers was associated with ineffective initial treatment. Of the 51 patients, seven (13.73%) received treatment in a large general hospital, eight (15.69%) received conservative treatment in the outpatient clinic of a community hospital, 23 (45.10%) received external application of Chinese herbs at home and 13 (25.49%) did not receive any treatment.

The pathological type was squamous cell carcinoma in 43 cases (84.31%), including 42 cases of well-differentiated squamous cell carcinoma (Broder's Grade I) and one case of moderately differentiated squamous cell carcinoma (Broder's Grade II), melanoma in six cases (11.76%), basal cell carcinoma in one case and epithelioid sarcoma in one case. The rate of metastasis varied among the pathological types. In patients with squamous cell carcinoma, the rate of sentinel lymph node metastasis was 30.23% and the rate of distant metastasis was 11.63%. In patients with melanoma, the rate of sentinel lymph node metastasis was 66.67% and the rate of distant metastasis was 33.33%. Lymph node and distant metastasis were not detected in the patients with basal cell carcinoma and epithelioid sarcoma (Table I).

The rates of lymph node metastasis and distant metastasis in patients with squamous cell carcinoma varied according to the location of the lesion. In patients with squamous cell carcinoma of the lower limb, the rate of sentinel lymph node metastasis was 35.48% and the rate of distant metastasis was 16.13%. In patients with squamous cell carcinoma of the upper limb, the rate of sentinel lymph node metastasis was 28.57% and the rate of distant metastasis was 0% (Table II). Eleven patients with squamous cell carcinoma and two patients with melanoma with deep, aggressive tumors and suspected sentinel lymph node metastasis underwent ^18^F-fluorodeoxyglucose PET-CT and B-mode ultrasound guided biopsy. These investigations had a 100% accuracy rate for the detection of metastasis (Table III).

#### Surgical methods and follow-up results

One patient with basal cell carcinoma on the head underwent extended resection and skin grafting, with no evidence of relapse or metastasis after eight years of follow-up. One patient with an epithelioid sarcoma over the occipital region underwent extended resection and skin grafting, with no evidence of relapse or metastasis after seven years of follow-up. Of the 43 patients with squamous cell carcinoma, 27 did not develop aggressive tumors or sentinel lymph node metastasis. These 27 patients underwent extended resection and skin grafting or skin flap repair. One of these patients succumbed to extensive metastasis after three years. Five patients developed deep, aggressive tumors with no metastasis. Four of these five patients underwent amputation and survived. One patient refused amputation and underwent only resection of the ulcer and surrounding tissues with skin grafting, and subsequently developed metastasis and succumbed one year later. Eleven patients developed deep, aggressive squamous cell carcinoma with inguinal, popliteal or axillary sentinel lymph node metastasis. Nine of these 11 patients underwent amputation and sentinel lymph node dissection, and eight patients survived. One patient who underwent amputation and inguinal lymph node dissection succumbed two years later due to pelvic lymph nodes and lung metastasis. One patient refused surgery and developed metastasis, and succumbed two years later. One patient developed deep, aggressive squamous cell carcinoma with extensive sentinel lymph node metastasis and distant pelvic lymph nodes metastasis. Radiotherapy was administered instead of surgery and the patient succumbed one year later due to lung metastasis (Table IV).

Two of the six patients with melanoma succumbed. One patient with melanoma on the right foot, right inguinal lymph node metastasis and lung metastasis was considered to have unresectable disease and received interferon therapy, and one patient with melanoma on the left foot and left inguinal lymph node metastasis refused surgery. These two patients succumbed from lung metastasis six months later. The other four patients survived. Two of these four patients did not develop metastasis, and underwent extended resection and skin grafting or skin flap reconstruction. The remaining two patients with melanoma on the foot and inguinal lymph node metastasis underwent extended resection and skin grafting or amputation combined with inguinal lymph node dissection, and survived with no evidence of relapse or metastasis (Table V).

**Figure 1.**
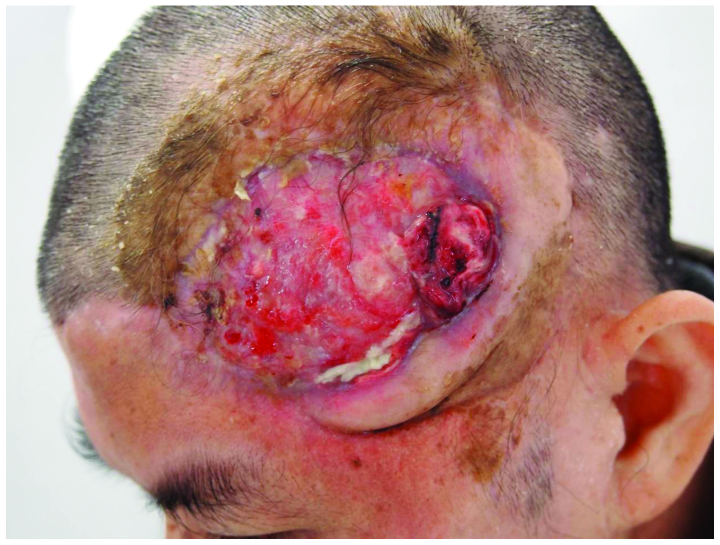
Squamous cell carcinoma arising in an ulcer on the head, showing invasion of the cranium and dura mater.

**Figure 2.**
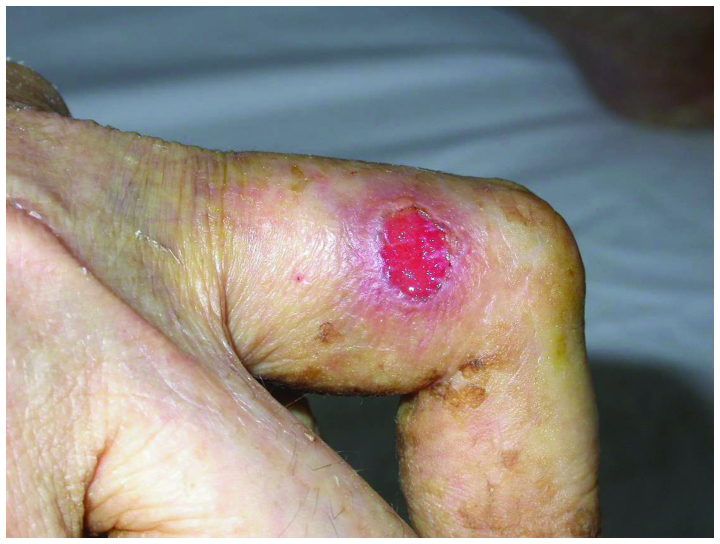
Well-differentiated squamous cell carcinoma arising in an ulcer on the left middle finger.

A number of patients had unusual presentations of disease. In one patient, squamous cell carcinoma developed simultaneously in traumatic skin ulcers on the lateral and medial sides of the left ankle. One patient developed squamous cell carcinoma in a burn scar ulcer over the temple, which extended through the bone and dura mater into the brain (Fig. 1). Three patients developed squamous cell carcinoma in an ulcer on the finger (Fig. 2).

#### Case report

A 55-year-old male with a 20-year history of ulceration over the lateral and medial aspects of his left ankle presented with a two-month history of pain. In 1993, he had developed chronic ulceration on either side of the ankle from friction caused by his shoe. The wounds were originally treated with saline irrigation by a rural doctor. The patient worked in a paddy field and had poor economic circumstances.

**Figure 3.**
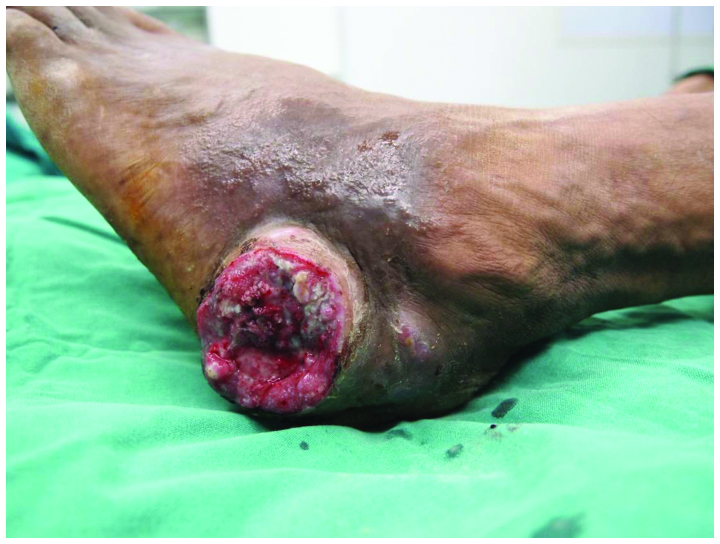
Well-differentiated squamous cell carcinoma arising in an ulcer on the lateral aspect of the left ankle in a patient with simultaneous carcinomatous ulcers on the medial and lateral aspects of the ankle.

**Figure 4.**
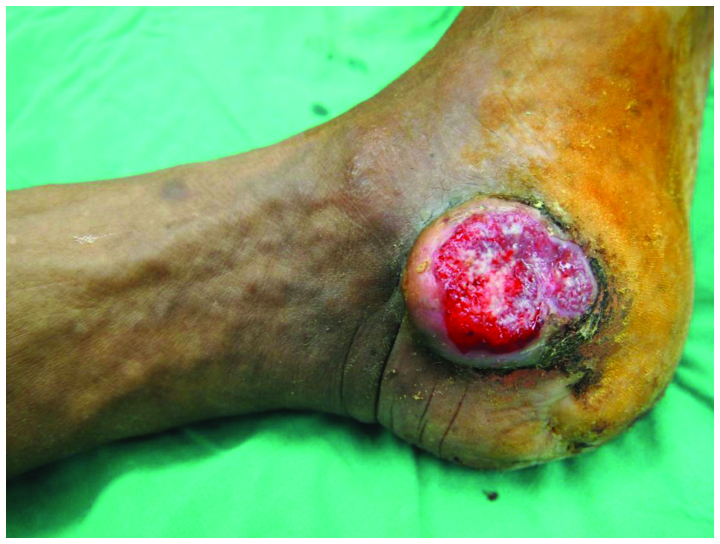
Well-differentiated squamous cell carcinoma arising in an ulcer on the medial aspect of the left ankle of the patient shown in Fig. 3.

Physical examination revealed a 4.5-cm-diameter ulcer over the medial aspect and a 5-cm-diameter ulcer over the lateral aspect of the left ankle. A Marjolin's ulcer with similar histological characteristics occurring in different parts of the body simultaneously is a rarely reported occurrence. The crater-shaped ulcers were dirty, necrotic and malodorous, with surrounding tissue proliferation (Figs. 3 and 4).

**Figure 5.**
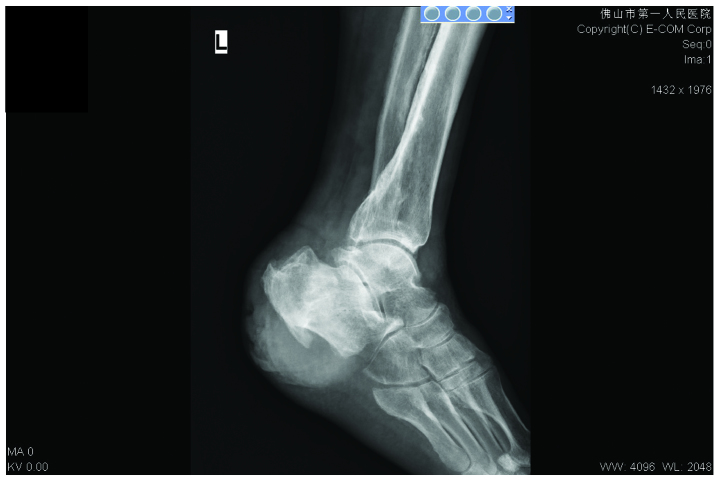
Radiographic findings from the patient shown in Fig. 3, showing bone changes and signs of osteomyelitis.

Radiography showed areas of dense cortical bone and new periosteal bone formation in the middle and distal parts of the left tibia and fibula, and in the calcaneus and talus. There was a small area of bone destruction in the distal part of the tibia, with signs of chronic osteomyelitis and surrounding soft tissue swelling (Fig. 5). Bacterial cultures of the wound surface revealed *Proteus penneri*.

**Figure 6.**
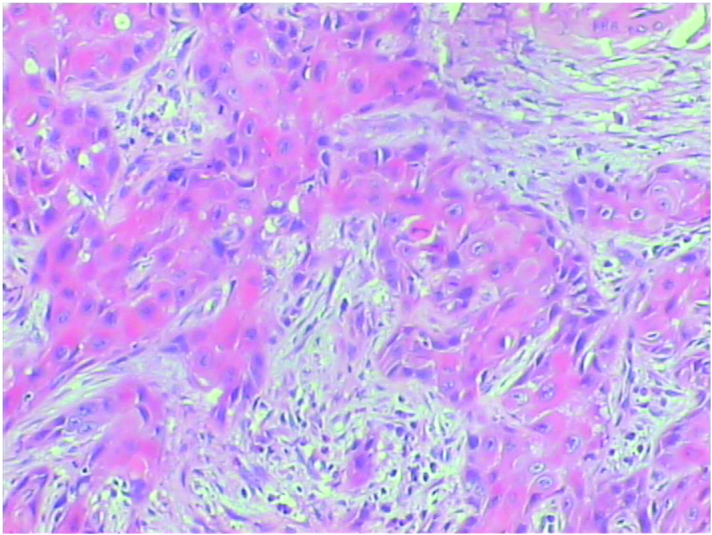
Pathological findings from the patient shown in Fig. 3, showing well-differentiated squamous cell carcinoma (stain, hematoxylin and eosin staining; magnification, x400).

**Figure 7.**
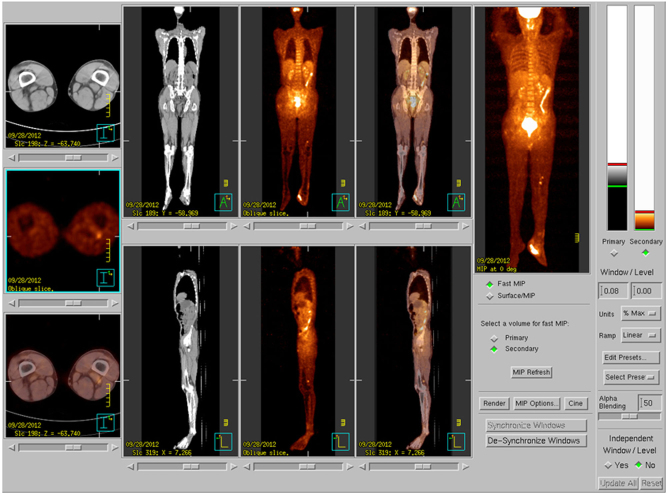
^18^F-Fluorodeoxyglucose-positron emission tomography findings from the patient shown in Fig. 3, showing increased uptake in the lymph nodes of the left popliteal fossa and left inguinal region.

In September 2012, the patient underwent partial resection of the lesions. Pathological examination showed well-differentiated squamous cell carcinoma in the two lesions (Fig. 6). PET-CT showed abnormal uptake in the lymph nodes of the left popliteal fossa and left inguinal region, but it was unclear whether this represented wound infection or tumor metastasis (Fig. 7).

**Figure 8.**
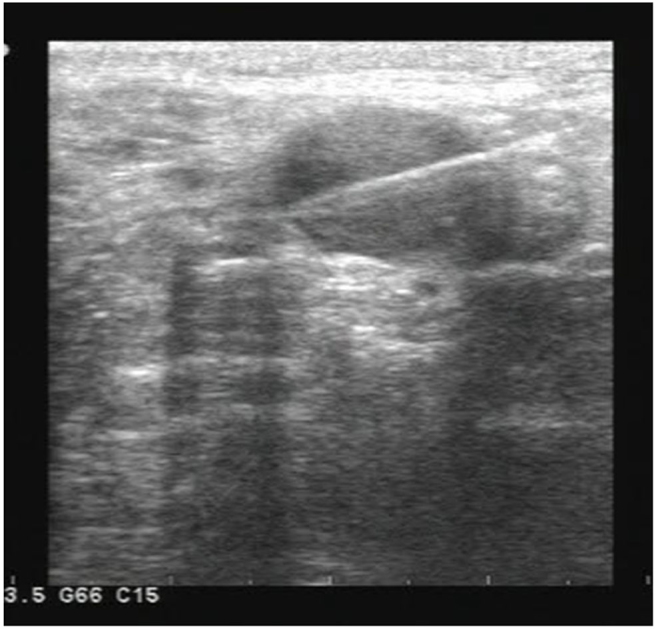
Ultrasound-guided biopsy findings of a left popliteal lymph node in the patient shown in Fig. 3.

In September 2012, the patient underwent below-knee amputation of the left leg for these aggressive lesions. Two weeks after surgery, PET-CT still showed increased uptake in the lymph nodes of the left popliteal fossa and inguinal region, indicating possible metastasis. The patient then underwent B-mode ultrasound-guided biopsy of the left popliteal and inguinal lymph nodes. Examination of the biopsy specimens showed metastasis in the popliteal nodes, but not in the inguinal nodes (Fig. 8).

The patient underwent left popliteal and inguinal lymph node dissection. Postoperative pathological examination showed metastatic squamous cell carcinoma in the popliteal nodes but not in the inguinal nodes, which was consistent with the previous biopsy findings. There was no evidence of relapse or metastasis after one year.

## Discussion

Marjolin's ulcers are tumors that form in chronic skin ulcers, predominantly on burn scar wounds. These tumors also develop on other wounds, including pressure sores (7), venous stasis ulcers (8), traumatic wounds (9), osteomyelitis (10), fistulas (11), leprosy ulcers (12) and lacerations (13). Burn scars are reported to have a rate of carcinomatous change of 2% (14). The most common type of Marjolin's ulcer is squamous cell carcinoma, followed by basal cell carcinoma, sarcoma and melanoma (15,16). Kowal-Vern and Criswell (17) retrospectively reviewed 412 cases of Marjolin's ulcers reported in 146 studies between 1923 and 2004, and found that 71% had squamous cell carcinoma, 12% had basal cell carcinoma, 6% had melanoma, 5% had sarcoma and 6% had other tumors. The present study included 51 patients with Marjolin's ulcers, including 43 (84.31%) with squamous cell carcinoma and six (11.76%) with melanoma.

Kowal-Vern and Criswell (17) reported that the average period of ulceration prior to carcinomatous change was 31 years. The present study included more female (56.86%) than male (43.14%) patients. The mean period of ulceration prior to carcinomatous change was relatively short (13.42 years for squamous cell carcinoma and 2.47 years for melanoma). The rates of lymph node and distant metastasis are higher in squamous cell carcinoma-type Marjolin's ulcer than in primary cutaneous squamous cell carcinoma (4,18). Kowal-Vern and Criswell (17) reported regional or sentinel lymph node metastasis in 22% of cases of squamous cell carcinoma-type Marjolin's ulcer, distant metastasis in 14% and a resulting mortality rate of 21%. Novick *et al* (19) reported a metastasis rate of 54% from lower limb squamous cell carcinoma-type Marjolin's ulcer, including metastases to the brain, liver, lung, kidney and distant lymph nodes. In the present study, patients with squamous cell carcinoma had a regional or sentinel lymph node metastasis rate of 30.23% and a distant metastasis rate of 11.63%. In patients with squamous cell carcinoma of the lower limb, the rate of sentinel lymph node metastasis was 35.48% and the rate of distant metastasis was 16.13%. In patients with squamous cell carcinoma of the upper limb, the rate of sentinel lymph node metastasis was 28.57% and the rate of distant metastasis was 0%. The location of the tumor was strongly associated with the rate of metastasis. Squamous cell carcinoma in the lower limb has previously been reported to have a higher rate of metastasis (20). Among patients with melanoma, 66.67% had sentinel lymph node metastasis and 33.33% had distant metastasis.

Squamous cell carcinoma and melanoma are aggressive types of tumor with high rates of metastasis. It is therefore important to detect sentinel lymph node and distant metastases prior to deciding the therapeutic regimen. Patients with sentinel lymph node metastasis should undergo lymph node dissection (21). PET-CT has a high sensitivity for the detection of metastasis and has been reported to be useful for the detection of lymph node metastasis in patients with malignant melanoma (22). Sentinel lymph node biopsy is a relatively non-traumatic method of screening for lymph node metastasis in patients with squamous cell carcinoma-type Marjolin's ulcers (23). In the present study, we were able to identify sentinel lymph node metastasis by detecting areas of increased uptake on PET-CT. However, B-mode ultrasound-guided biopsy and surgical specimen examination findings showed that certain nodes with increased uptake on PET-CT exhibited inflammatory hyperplasia but not metastasis. The reasons for this are unclear. PET-CT findings alone are therefore insufficient for the definitive diagnosis of lymph node metastasis, and they should be used in combination with ultrasound-guided biopsy findings. The Affiliated Foshan Hospital started using a Philips Gemini PET-CT scanner (Philips Healthcare, Best, the Netherlands) in February 2004. In the present study, only 11 patients underwent both PET-CT and ultrasound guided biopsy, and the accuracy rate for diagnosis of sentinel lymph node metastasis was 100% in these patients. Prior to the introduction of PET-CT, patients with suspected sentinel lymph node metastasis underwent B-mode ultrasound and CT examinations, but the findings were less precise than those with PET-CT. Distant metastasis can be detected early using PET-CT alone, and patients with distant metastasis are considered to have unresectable disease.

The pathogenesis of Marjolin's ulcers remains poorly understood. Development of squamous cell carcinoma in burn scar ulcers was reported to be associated with local *Fas* gene mutation and deletion (24,25). Diagnosis of Marjolin's ulcers depends on the pathological examination of biopsy specimens. Sampling from different sites increases the diagnostic rate (16). Patients with chronic or recurrent skin ulcers that do not heal after several months of conservative treatment should undergo biopsy for early diagnosis. Marjolin's ulcers should be treated by extended resection and skin grafting or skin flap repair (26). The resection margin should extend ≥2 cm beyond the edges of the lesion (20). Amputation is necessary when the tumor has invaded the bones, for aggressive tumors and for tumors that cannot otherwise be resected with adequate margins. Sentinel lymph node dissection is required in patients with sentinel lymph node metastasis (9,20,26). Patients with squamous cell carcinoma and sentinel lymph node metastasis can undergo amputation and sentinel lymph node dissection. The present data confirm that squamous cell carcinoma-type Marjolin's ulcers can occur in different regions of the body, but that sentinel lymph node metastasis most commonly occurs in limb lesions, particularly of the lower limb. Patients with limb lesions can therefore be treated by amputation and sentinel lymph node dissection with satisfactory results.

Similar to patients with squamous cell carcinoma, patients with melanoma who do not have metastasis should undergo more extended resection and skin grafting or skin flap repair. Patients with sentinel lymph node metastasis but no distant metastasis should undergo amputation with lymph node dissection. In the present study, all malignant melanoma-type ulcers occurred in the lower limb. However, unlike with squamous cell carcinoma, patients with melanoma and with distant or extensive lymph node metastasis cannot be cured by surgical treatment, and interferon therapy should be considered in these patients, despite its poor curative effects.

There is no evidence that radiotherapy is a successful first-line treatment choice for squamous cell carcinoma. Squamous cell carcinoma in Marjolin's ulcers is usually well- or moderately differentiated, and radiotherapy is therefore not effective (16,21). Radiotherapy may also induce further carcinomatous change. Radiotherapy was therefore not selected as the first treatment choice in any of the patients in this study.

Marjolin's ulcers are preventable. Chronic skin ulcers should be actively treated to avoid carcinomatous change (26,27). In this study, the mean patient age was 64.15 years. The majority of the patients had been treated ineffectively with Chinese herbs or other local remedies due to their poor financial circumstances, and some did not receive any treatment. This resulted in chronic ulceration that eventually underwent carcinomatous change. Recently, a new cooperative medical care system has been developed in rural areas of China, and the Urban Employee Medical Insurance system has been established (28,29). Patients with financial restrictions can therefore be treated in hospital, which may help to reduce the incidence of Marjolin's ulcers.

In conclusion, the results of the present study strongly indicate that chronic skin ulcers should be treated as early as possible and carefully followed-up. PET-CT combined with B-mode ultrasound-guided biopsy can precisely detect sentinel lymph node metastasis and guide clinical therapy. Patients with squamous cell carcinoma- or melanoma-type Marjolin's ulcers and sentinel lymph node metastasis should undergo amputation and sentinel lymph node dissection, since such tumors predominantly occur in the limb, particularly in the lower limb.

## Figures and Tables

**Table I.** Metastasis according to the pathological type of Marjolin's ulcer.

Pathological type	n	Patients with sentinel lymph node metastasis, n (%)	Patients with distant metastasis, n (%)
Squamous cell carcinoma	43	13 (30.23)	5 (11.63)
Melanoma	6	4 (66.67)	2 (33.33)
Basal cell carcinoma	1	0 (0.00)	0 (0.00)
Epithelioid sarcoma	1	0 (0.00)	0 (0.00)

**Table II.** Sentinel lymph node and distant metastases according to the location of squamous cell carcinoma.

Location	n	Sentinel lymph node metastasis, n (%)	Distant metastasis, n (%)
Lower limb	31	11 (35.48)	5 (16.13)
Upper limb	7	2 (28.57)	0 (0.00)
Head	4	0 (0.00)	0 (0.00)
Chest	1	0 (0.00)	0 (0.00)

**Table III.** PET-CT and B-mode ultrasound-guided biopsy findings in 11 patients with aggressive tumors and suspected lymph node metastasis.

Case no.	Gender	Age (years)	Site of tumor	Type of tumor	Region of lymph node metastasis indicated by PET-CT	Results of biopsy under ultrasound B-mode
1	Female	47	Left toes	Well-differentiated SCC	Left groin	Positive
2	Female	37	Left foot	Moderately differentiated SCC	Left/right groin	Positive/positive
3	Male	47	Head	Well-differentiated SCC	Head	Negative
4	Female	83	Left foot	Well-differentiated SCC	Left groin	Positive
5	Male	55	Left ankle	Well-differentiated SCC	Left groin	Negative
					Left popliteal	Positive
6	Male	57	Right foot	Melanoma	Right groin	Positive
7	Male	54	Left forearm	Well-differentiated SCC	Left axillary	Positive
8	Male	64	Right popliteal fossa	Well-differentiated SCC	Right groin	Positive
9	Female	47	Right hand	Well-differentiated SCC	Right axillary	Positive
10	Male	48	Left toe	Well-differentiated SCC	Left groin	Positive
11	Male	53	Right foot	Melanoma	Left groin	Positive

SCC, squamous cell carcinoma; PET-CT, positron emission tomography-computed tomography.

**Table IV.** Treatment and follow-up results in 43 patients with squamous cell carcinoma.

Case no.	Age (years)	Tumor location	Tumor aggression, lymphatic metastasis and distant metastasis	Surgical method	Follow-up (years)	Follow-up results
1	75	Left calf	No	Extended resection, skin grafting	8	Presence
2	53	Right foot	No	Extended resection, skin grafting	7	Presence
3	48	Scalp	No	Extended resection, local skin flap	5	Presence
4	32	Right popliteal fossa	No	Extended resection, skin grafting	3	Presence
5	41	Left thigh	No	Extended resection, skin grafting	2	Presence
6	56	Left calf	No	Extended resection, skin grafting	1	Presence
7	73	Right popliteal fossa	No	Extended resection, skin grafting	2	Presence
8	62	Left foot	No	Extended resection, skin grafting	4	Presence
9	37	Left elbow	No	Extended resection, skin grafting	2	Presence
10	78	Left forearm	No	Extended resection, skin grafting	3	Presence
11	56	Right popliteal fossa	No	Extended resection, axial skin flap	2	Presence
12	47	Left calf	No	Extended resection, Free skin flap	3	Presence
13	89	Right foot	No	Extended resection, skin grafting	4	Presence
14	75	Left foot	No	Extended resection, skin grafting	2	Presence
15	51	Left popliteal fossa	No	Extended resection, skin grafting	8	Presence
16	61	Left thigh	No	Extended resection, skin grafting	2	Presence
17	63	Right foot	No	Extended resection, skin grafting	2	Presence
18	47	Right foot	No	Extended resection, skin grafting	5	Presence
19	61	Left thigh	No	Extended resection, skin grafting	2	Presence
20	75	Left foot	No	Extended resection, skin grafting	1	Presence
21	68	Left middle finger	No	Extended resection, skin grafting	7	Presence
22	47	Scalp	No	Extended resection, local skin flap	1	Presence
23	57	Chest	No	Extended resection, skin grafting	3	Presence
24	75	Scalp	No	Extended resection, skin grafting	1	Presence
25	46	Scalp	No	Extended resection, free skin flap	4	Presence
26	67	Right eyelid	No	Extended resection, local skin flap	7	Presence
27	37	Left foot	No	Extended resection, axial skin flap	3	Metastasized to bilateral inguinal groove, left thigh and lung; mortality
28	58	Right footplate	Deep aggression, no metastasis	Amputation	8	Presence
29	74	Right forefinger	Deep aggression, no metastasis	Finger amputation	1	Presence
30	79	Right forefinger	Deep aggression, no metastasis	Finger amputation	3	Presence
31	38	Left footplate	Deep aggression, no metastasis	Amputation	2	Presence
32	89	Left popliteal fossa	Deep aggression, no metastasis	Refused to amputation; extended resection, skin grafting only	1	Metastasized to left inguinal lymph node and lung; mortality
33	64	Right popliteal fossa	Deep aggression; metastasized to right inguinal lymph node	Amputation, right inguinal lymph node dissection	5	Presence
34	48	Left toe	Deep aggression; metastasized to left inguinal lymph node	Toe amputation, left inguinal lymph node dissection	2	Presence
35	55	Left ankle	Deep aggression; metastasized to left popliteal lymph node	Amputation, left popliteal and inguinal lymph node dissection	1	Presence
36	66	Right foot	Deep aggression; metastasized to right inguinal lymph node	Amputation, right inguinal lymph node dissection	5	Presence
37	47	Right hand	Deep aggression; metastasized to left axillary lymph node	Amputation, left axillary lymph node dissection	2	Presence
38	54	Left forearm	Deep aggression; metastasized to left axillary lymph node	Amputation, left axillary lymph node dissection	4	Presence
39	51	Right popliteal fossa	Deep aggression; metastasized to left inguinal lymph node	Amputation, left inguinal lymph node dissection	1	Presence
40	47	Left toe	Deep aggression; metastasized to left inguinal lymph node	Amputation, left inguinal lymph node dissection	2	Presence
41	58	Left popliteal fossa	Deep aggression; metastasized to left inguinal lymph node	Amputation, left inguinal lymph node dissection	2	Metastasized to pelvic lymph nodes and lung; mortality
42	83	Left foot	Deep aggression; metastasized to left inguinal lymph node	Refused to undergo surgery	2	Metastasized to lung; mortality
43	78	Left thigh	Deep aggression; metastasized to left inguinal lymph nodes and pelvic lymph nodes	No surgery, radiotherapy	1	Metastasized to lung; mortality

**Table V.** Characteristics of six patients with melanoma.

Case no.	Tumor location	Lymphatic and distant metastases	Therapy	Follow-up (months)	Follow-up results
1	Right foot	Lung, right inguinal lymph node metastasis	Interferon	6	Lung metastasis; mortality
2	Left footplate	Left inguinal lymph node metastasis	Refused the treatment	6	Lung metastasis; mortality
3	Left footplate	No metastasis	Extended resection, skin grafting	21	Presence
4	Left footplate	No metastasis	Extended resection, medial pedal flap of footplate	41	Presence
5	Right footplate	Right inguinal lymph node metastasis	Amputation, right inguinal lymph node dissection	31	Presence
6	Right heel	Right inguinal lymph node metastasis	Extended resection, skin grafting, right inguinal lymph node dissection	26	Presence
